# Changes in intestinal homeostasis and immunity in a cigarette smoke- and LPS-induced murine model for COPD: the lung-gut axis

**DOI:** 10.1152/ajplung.00486.2021

**Published:** 2022-06-14

**Authors:** Lei Wang, Charlotte E. Pelgrim, Lucía N. Peralta Marzal, Stephanie Korver, Ingrid van Ark, Thea Leusink-Muis, Ardy van Helvoort, Ali Keshavarzian, Aletta D. Kraneveld, Johan Garssen, Paul A. J. Henricks, Gert Folkerts, Saskia Braber

**Affiliations:** ^1^Division of Pharmacology, Utrecht Institute for Pharmaceutical Sciences, Faculty of Science, Utrecht University, Utrecht, The Netherlands; ^2^Danone Nutricia Research, Utrecht, The Netherlands; ^3^NUTRIM School of Nutrition and Translational Research in Metabolism, Maastricht University, Maastricht, The Netherlands; ^4^Division of Digestive Diseases and Nutrition, Department of Internal Medicine, Rush Medical College, Rush University, Chicago, Illinois

**Keywords:** cigarette smoke exposure, COPD, gut-lung axis, intestinal immunity, systemic inflammation

## Abstract

Chronic obstructive pulmonary disease (COPD) is often associated with intestinal comorbidities. In this study, changes in intestinal homeostasis and immunity in a cigarette smoke (CS)- and lipopolysaccharide (LPS)-induced COPD model were investigated. Mice were exposed to cigarette smoke or air for 72 days, except *days 42*, *52*, and *62* on which the mice were treated with saline or LPS via intratracheal instillation. Cigarette smoke exposure increased the airway inflammatory cell numbers, mucus production, and different inflammatory mediators, including C-reactive protein (CRP) and keratinocyte-derived chemokine (KC), in bronchoalveolar lavage (BAL) fluid and serum. LPS did not further impact airway inflammatory cell numbers or mucus production but decreased inflammatory mediator levels in BAL fluid. T helper (Th) 1 cells were enhanced in the spleen after cigarette smoke exposure; however, in combination with LPS, cigarette exposure caused an increase in Th1 and Th2 cells. Histomorphological changes were observed in the proximal small intestine after cigarette smoke exposure, and addition of LPS had no effect. Cigarette smoke activated the intestinal immune network for IgA production in the distal small intestine that was associated with increased fecal sIgA levels and enlargement of Peyer’s patches. Cigarette smoke plus LPS decreased fecal sIgA levels and the size of Peyer’s patches. In conclusion, cigarette smoke with or without LPS affects intestinal health as observed by changes in intestinal histomorphology and immune network for IgA production. Elevated systemic mediators might play a role in the lung-gut cross talk. These findings contribute to a better understanding of intestinal disorders related to COPD.

## INTRODUCTION

Chronic obstructive pulmonary disease (COPD) is one of the leading causes of morbidity and mortality worldwide and is characterized by irreversible alterations to the structure of the lungs and chronic inflammation in pulmonary tissues (1). COPD is also a multifactorial systemic disease and the chronic inflammation observed in the lungs of patients with COPD is associated with systemic effects, such as mental (2), cardiovascular, metabolic, musculoskeletal, and gastrointestinal comorbidities (3). Patients with COPD often have a high prevalence and incidence of intestinal symptoms such as inflammatory infiltration, increased intestinal permeability, and absorptive impairment (4–6). For example, patients with COPD have an increased risk of developing inflammatory bowel disease (IBD) (7, 8), and this risk increases with the severity of COPD (9). COPD and IBD share many similarities in epidemiological and clinical features, as well as in inflammatory responses. Dysregulation of immunity at mucosal surfaces is thought to be responsible for the development and progression of COPD and IBD (10). It has been hypothesized that the airway inflammation observed in COPD results in enhanced systemic inflammation (11). Systemic inflammation may be a central pathogenic link between the pulmonary and extrapulmonary components, like the gastrointestinal tract (12). The systemic impact of COPD further increases the risk of morbidities and mortalities (13, 14).

The bidirectional cross talk between the gut and the lungs with their shared mucosal immune system has been called the gut-lung axis (15). During inflammation, the bronchus-associated lymphoid tissue (BALT) controls immune cell trafficking from the lung tissue into the systemic circulation, which reflects the role of the gut-associated lymphoid tissue (GALT). These immune cells from lung and intestinal tissue exhibit the ability to migrate to the other mucosal sites (16, 17). Immunoglobulin A (IgA) is one of the key antibodies in mucosal tissues and seems to be essential to establish a balanced and healthy intestinal mucosal immune response to protect against invading pathogens, but also to influence mucosal tolerance toward safe food proteins/antigens (18, 19). In addition, systemic mediators, dysregulation of protease activity, and interaction of the lung and intestinal microbiome might play an important role in the gut-lung cross talk (17).

Cigarette smoke is one of the most prominent risk factors for developing COPD (20). Epidemiologic studies indicated that there are shared risk factors, such as cigarette smoking and an unhealthy lifestyle (e.g., diet) for the development of respiratory diseases, such as COPD and gastrointestinal complaints (21). Besides the effects of cigarette smoke on the development of COPD, respiratory bacterial, viral, and/or fungi infections are also major risk factors potentially exacerbating symptoms of COPD and resulting in hospitalization and additional treatment (22, 23). It is well established that additional exposure to the bacterial endotoxin lipopolysaccharide (LPS) in cigarette smoke- or elastase-induced animal models for COPD can cause an exaggerated inflammatory response in the lungs (24, 25). Intestinal changes in cigarette smoke-exposed mice following repetitive exacerbations have not yet been studied so far. Therefore, a murine model of long-term cigarette smoke exposure with or without LPS costimulation was used to better understand the effects of pulmonary inflammation and emphysema on systemic inflammation and intestinal homeostasis and immunity. In this perspective, inflammatory mediators in bronchoalveolar lavage (BAL) fluid and lung histology were investigated to confirm that cigarette smoke with or without LPS exposure induced lung injury. Systemic inflammation was confirmed by measuring inflammatory mediators in serum and T cell subsets in spleen. The impact of cigarette smoke with or without LPS exposure on intestinal homeostasis was studied by exploring intestinal histomorphology, the short-chain fatty acids (SCFAs) production in the gut, and the intestinal immune network for IgA production. We hypothesize that cigarette smoke exposure might affect the immunity of the gut and induce intestinal damage besides the observed lung injury, and the additional LPS triggers may influence the cigarette smoke-induced immune modulation within the gut-lung axis.

## MATERIALS AND METHODS

### Animals

Specific pathogen-free female Balb/c mice, 11–13 wk old, were obtained from Charles River Laboratories (26, 27). Balb/c mice show a high neutrophil influx in the lungs after exposure to cigarette smoke as compared with other mouse strains (28). Mice were housed in groups (3 or 4 animals/cage) in filter-topped makrolon cages (22 cm × 16 cm × 14 cm, floor area 350 cm^2^, Tecnilab-BMI, Someren, The Netherlands) with wood-chip bedding (Tecnilab-BMI, Someren, The Netherlands) and tissues (VWR, The Netherlands) were available as cage enrichment. Mice were kept under standard conditions on a 12-h light-dark cycle (lights on from 7:00 AM to 7:00 PM) at controlled relative humidity (relative humidity of 50%–55%) and temperature (21 ± 2°C) at the animal facility of Utrecht University.

Food (AIN-93M, SNIFF Spezialdiäten GmbH, Soest, Germany) and water were refreshed once a week. Based on average body weight, mice were divided into four experimental groups (*n* = 15/16 animals per group): air exposure group, LPS treatment group, cigarette smoke exposure group, and cigarette smoke exposure plus LPS treatment group. All animal procedures described in this study were approved by the Ethics Committee of Animal Research of Utrecht University, Utrecht, The Netherlands (AVD1080020184785) and were conducted in accordance with governmental guidelines.

### Murine COPD Model

**Figure 1.**
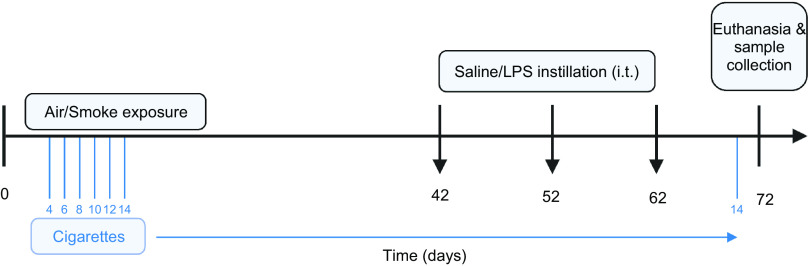
Experimental design. Mice were exposed to air or smoke for 72 days by using 4 cigarettes on *day 1*; 6 cigarettes on *day 2*; 8 cigarettes on *day 3*; 10 cigarettes on *day 4*; 12 cigarettes on *day 5*; and 14 cigarettes from *day 6* until the end of the study. Mice were not exposed to air or smoke on *days 42*, *52*, and *62*, but were treated with saline or lipopolysaccharide (LPS) via intratracheal (it) instillation on these days. On *day 72*, mice were euthanized and samples were collected for future analysis.

Mice were exposed in whole body chambers to mainstream cigarette smoke or air using a peristaltic pump (SCIQ 232, Watson-Marlow 323). A Plexiglas box containing four metal cages each with four compartments was used to expose the mice to either cigarette smoke or air. Two mice from the same home cage were placed in each compartment. Research cigarettes (3R4F) were obtained from the Tobacco Research Institute (University of Kentucky, Lexington, KY) (29) and filters were removed before use (26). Mice were exposed to cigarette smoke once a day for 72 consecutive days (except on *days 42*, *52*, and *62*). Mice were acclimatized to cigarette smoke exposure by gradually increasing the number of cigarettes during the first days of the experiment using 4 cigarettes on *day 1*; 6 cigarettes on *day 2*; 8 cigarettes on *day 3*; 10 cigarettes on *day 4*; 12 cigarettes on *day 5*; and 14 cigarettes from *day 6* until the end of the study. LPS (50 μL of 10 μg/mL) (*Escherichia coli* O55:B5, Sigma Aldrich, Missouri) (30) or vehicle (saline) was administrated via intratracheal instillation (it) under isoflurane anesthesia (28) on *days 42*, *52*, and *62* instead of being exposed to air or cigarette smoke (Fig. 1) (31). For practical reasons, one dose of LPS, 10 μg/mL, was selected based on the results of Pelgrim et al. (32). The smoke chamber was connected to a peristaltic pump and vacuum to produce smoke and control the air circulation. The speed of the pump was kept at 35 rpm and the CO levels ranged between 200 and 400 ppm. The mass concentration of cigarette smoke total particulate matter (TPM) was determined by gravimetric analysis of type A/E glass fiber filter (PALL life sciences, Mexico). The TPM concentration in the smoke exposure box generated by 14 cigarettes reached ∼828 μg/L (828 ± 4.5 μg/L) (26).

Mice were anesthetized by an intraperitoneal injection of ketamine/medetomidine (196.8 mg/kg and 1.32 mg/kg respectively) ±18 h after the last air or smoke exposure. Blood was obtained and collected in Mini collect tubes (Greiner bio-one, Alphen aan den Rijn, the Netherlands). Blood samples were centrifuged (14,000 *g* for 10 min) and serum was stored at −20°C. Feces and cecum content were collected, snap-frozen in liquid nitrogen, and kept at −80°C until further analysis. Lungs, proximal and distal small intestine from half of the animals in each group (*n* = 7 or 8/group) were collected and partly used for histology. The proximal and distal small intestines from the other half of the animals in each group (*n* = 7 or 8/group) were snap-frozen in liquid nitrogen and kept at −80°C until RNA sequencing analysis (*n* = 3 or 5/group). Spleens were used for the fluorescence-activated cell sorting (FACS) analysis (*n* = 15/group).

### Tissue Preparation for Histological Analysis

Lungs were perfused and fixed with 10% formalin at a constant pressure. The proximal small intestine (duodenum) and distal small intestine (ileum) were dissected. Fecal matter was removed by gentle flushing with phosphate-buffered saline (PBS), then “swiss rolls” were obtained and fixed in 10% formalin. Tissues were embedded in paraffin (Tissue processor, Leica), 5 μm sections of the lung and intestinal tissue were cut using a microtome (Leica RM 2165) and mounted on glass slides (StarFrost adhesive slides, Knittelgläser, Germany). Slides were deparaffinized and used for hematoxylin and eosin (H&E) and Alcian blue-periodic acid-Schiff (AB-PAS) staining according to the standard protocols. Microscopic images were taken using an Olympus BX50 microscope (Olympus, Tokyo, Japan). The inflammatory cells and mucin-containing cells were evaluated around the airways at 800 μm depth (2 or 3 images/animal) and the mean linear intercept (*L*_m_) was quantified using the lung tissue slides at 200 μm depth (6 images/animal) (32). The villus length and crypt depth were measured in the proximal and distal small intestine (7–9 villi and crypts per animal) and an example of the histomorphometric analysis of a villus and crypt is depicted in Supplemental Fig. S3. The size of the Peyer’s patches (2 or 3 Peyer’s patches/animal) was quantified in the distal small intestine. Seven or eight animals per group for both lung and intestine were evaluated. Histological slides of the lungs and intestines were examined blind in treatment group using ImageJ software [National Institutes of Health (NIH), Bethesda, MD].

### BAL Fluid Collection and Analysis

The trachea was exposed and a cannula was inserted in the trachea after making a small incision. BAL fluid was collected by lung lavage with 1 mL pyrogen-free saline (0.9% NaCl, 37°C) supplemented with protease inhibitors (Complete Mini, EDTA-free Protease Inhibitor Cocktail, Sigma Aldrich, Germany). This step was repeated three times with 1 mL pyrogen-free saline. The BAL fluid was centrifuged (400 *g* at 4°C for 5 min) and the supernatant of the first 1 mL was stored at −20°C for ELISA measurement and a Luminex assay.

### Inflammatory Mediators Measurement in Serum and BAL Fluid

Concentrations of inflammatory mediators, keratinocyte-derived chemokine (KC), IL-12, VEGF-A, IL-10, and IL-6 in the BAL fluid, and KC in serum were determined by a quantitative Milliplex Luminex assay kit (ProcartaPlex, Thermo Fisher Scientific, Austria) according to the manufacturer’s instructions and using Luminex 200TM with Xponent software. C-reactive protein (CRP) levels in the BAL fluid and serum were measured by ELISA (Mouse CRP ELISA kit, R&D Systems) according to the manufacturer’s instructions and using a microplate reader (Glomax Discover, Promega).

### SCFAs Concentrations

Levels of the SCFAs: acetic acid, propionic acid, butyric acid, iso-butyric acid, valeric acid, and iso-valeric acid in the cecum were quantified using direct-injection gas chromatography as described before (33, 34).

#### Preparations of cecum samples.

Cecal contents were snap-frozen in liquid nitrogen and stored at −80°C until measurement. SCFA samples were prepared based on the study by Thiel and Blaut (35). The cecum content was weighed (30–60 mg) and diluted by 10× cold PBS (wt/vol) in a new 1.5 mL Eppendorf tube, followed by adding 3-mm glass beads (Sigma Aldrich). Samples were homogenized by vortexing for 5 min. To remove glass beads and large particles, the samples were centrifuged at 300 *g* for 1 min and the supernatant was transferred to a 1.5-mL Eppendorf tube. This was followed by centrifugation for 10 min at 15,000 *g* (4°C). The created supernatant (200 µL) was loaded into a 96-wells plate and stored at −80°C for downstream analysis.

#### SCFAs analysis.

The SCFAs acetic, propionic, butyric, isobutyric, valeric, and isovaleric acids were quantitatively determined using a Shimadzu GC2025 gas chromatograph (Shimadzu Corporation, Kyoto, Japan) equipped with a flame ionization detector. The sample (0.5 µL) was injected at 80°C into the column (Stabilwax, 15 m × 0.53 mm, film thickness 1.00 µm; Restek Co., Bellafonte, PA) using H_2_ as carrier gas (20.7 kPa). New columns were conditioned overnight at 200°C. After injection of the sample, the oven was heated to 160°C at a rate of 16 °C/min, followed by heating to 220°C at 20 °C/min and finally maintained at a temperature of 220°C for 1.5 min. The temperature of the injector and the detector was 200°C. After every 10 samples, the column was cleared by injection of 0.5 µL formic acid (1%, by vol) to avoid memory effects of the column, followed by injection of 0.5 mL standard SCFA mix (1.77 mM acetic acid, 1.15 mM propionic acid, 0.72 mM butyric acid, 0.72 mM isobutyric acid, 0.62 mM valeric acid, and 0.62 mM isovaleric acid; Sigma Aldrich) to monitor the occurrence of memory effects. SCFA concentrations were determined using 2-ethylbutyric acid as an internal standard.

### RNA Isolation and RNA-Sequencing

#### RNA preparation.

Total RNA was isolated and extracted from proximal and distal small intestine tissue using the RNeasy Mini Kit according to the manufacturer’s protocol (Qiagen, Germany). RNA integrity and quantitation were assessed using the RNA Nano 6000 Assay Kit of the Bioanalyzer 2100 system (Agilent Technologies, CA). RNA degradation and contamination were monitored on 1% agarose gels.

#### mRNA nondirectional (polyA).

RNA sample was used for library preparation using NEB Next Ultra RNA Library Prep Kit for Illumina Indices were included to multiplex multiple samples. Briefly, mRNA was purified from total RNA using poly-T oligo-attached magnetic beads. After fragmentation, the first strand cDNA was synthesized using random hexamer primers followed by the second strand cDNA synthesis. The library was ready after end repair, A-tailing, adapter ligation, and size selection. After amplification and purification, insert size of the library was validated on an Agilent 2100 and quantified using quantitative PCR (Q-PCR). Libraries were then sequenced on Illumina NovaSeq 6000 S4 flowcell with PE150 according to results from library quality control and expected data volume. Library preparation/sequencing/analysis is performed by Novogene (UK) Company Limited.

### IgA Measurement in the Feces

Fecal samples were collected by placing each mouse in a separate clean box for 3–5 min 1 day before euthanization, and freshly defecated fecal pellets, uncontaminated with urine, were sampled, snap-frozen in liquid nitrogen, and stored at −80°C till further analysis. The wet weight of feces samples ranged from 10 to 32 mg (median: 17.5 mg). Extraction buffer [PBS (pH = 7.4), protease inhibitor cocktail (Sigma Aldrich, Germany), and 0.01% sodium azide] was added to each sample at a ratio of 1 mL buffer to 1 g feces. Samples were thoroughly homogenized by a homogenizer (Bertin Technologies, France) at 6,000 *g* for 20 s. Fecal suspensions were centrifuged at 14,000 *g* for 10 min at 4°C and stored at −20°C until further use. The optimum sample dilution was tested, and 200 times dilution was selected for the IgA measurement using an ELISA kit (Mouse IgA ELISA kit, Thermo Fisher Scientific, The Netherlands) according to the manufacturer’s instructions.

### Immunofluorescent Staining in Distal Small Intestine

The 5-μm formalin-fixed, paraffin-embedded distal small intestinal sections on glass slides were deparaffinized, rehydrated in decreasing concentrations of ethanol, and incubated with 0.3% H_2_O_2_/methanol for 30 min to quench endogenous peroxidase activity. Thereafter, the slides were incubated with rabbit-anti-IgA primary antibody (1:12,000; Novus Biologicals) at room temperature for 2 h after blocking with 5% goat serum in PBS containing 1% bovine serum albumin (BSA). After three washing steps with PBS containing 0.2% Tween-20 (PBST; pH 7.4), slides were incubated with Alexa fluorescently conjugated goat anti-rabbit secondary antibody (1:200; Invitrogen, The Netherlands) for 1 h at room temperature. The nuclei were stained by Hoechst (1:2,000; Invitrogen), slides were rinsed after the Hoechst staining, and mounted with FluorSave reagent (Merck Millipore, St. Louis, MO). Images were captured by the confocal microscope (TCS SP8 X, Leica, Germany).

### Fluorescence-Activated Cell Sorting

#### Cell isolation from tissues.

Spleens were crushed through 70-µm cell strainers. The splenocyte suspension was incubated with lysis buffer (8.3 g NH_4_Cl, 1 g KHCO_3_, and 37.2 mg EDTA dissolved in 1 L demi water and filter sterilized) to remove red blood cells and then resuspended in RPMI 1640 (Lonza, Basel, Switzerland) supplemented with 10% fetal bovine serum.

#### Flow cytometry of immune cells.

Spleen cells were resuspended in PBS-1% bovine serum albumin and incubated with anti-mouse CD16/CD32 (1:100, BD Fc Block) to block nonspecific binding sites. For surface staining, cells were incubated with CD4-PerCp-Cy5.5, CD69-APC, CXCR3-PE, (1:400, 1:100, and 1:100, eBiosciences), T1ST2-FITC, (1:200, MD Biosciences), CD4-Brilliant, CD196 (CCR6)-PE (1:160, 1:640, BioLegend), CD25-PerCp-Cy5.5 (1:1,280, Thermo Fisher), and CD127-PE-Cy7 (1:320, Miltenyi Biotec, Germany). Viable cells were distinguished by means of a fixable viability dye APC-Cy7 (1:2,000, eBioscience). For detecting transcription factors, cells were first fixed and permeabilized with Foxp3 Staining Buffer Set (eBioscience) according to the manufacturer’s protocol and then stained with FoxP3-FITC (1:100, Thermo Fisher) and RorgT-APC (1:400, Biosciences). The specificity of all antibodies was assessed and optimal working dilutions were determined in a previously published study from our group (36).

Results were collected with BD FACS Canto II flow cytometer (Becton Dickinson) and analyzed with FlowLogic software (Inivai Technologies, Australia). The gating strategies are shown in Supplemental Fig. S2.

### Statistical Analysis

All results are presented as means ± SE. Differences between groups were statistically determined by two-way ANOVA followed by Šidák’s multiple-comparisons test. Spearman tests were conducted for analyses of correlation. The difference was considered statistically significant at *P* < 0.05, and trends were considered when 0.05 < *P* < 0.1. All statistical analyses were performed using GraphPad Prism (version 8.3.0).

## RESULTS

### Evaluation of Lung Damage

#### Lung emphysema, inflammation, and mucus production.

**Figure 2.**
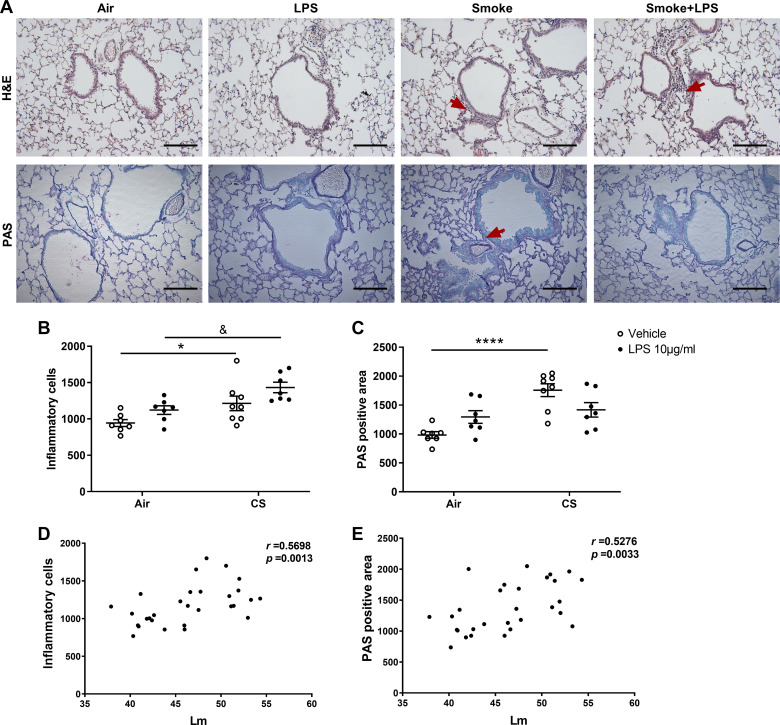
Lung emphysema, inflammation, and mucus production. Mice were exposed to air or cigarette smoke for 72 days, except on *days 42*, *52*, and *62*. On these days mice were treated with saline or lipopolysaccharide (LPS) via intratracheal (it) instillation. Lung tissue was collected and stained with hematoxylin and eosin (H&E) (*A*, *top*) and periodic acid-Schiff (PAS) (*A*, *bottom*) (800 μm depth) for each treatment group (magnification, ×200, Scale bar = 200 μm). Inflammatory cells (*B*) and PAS positive cells (*C*) were analyzed by ImageJ. Correlations between inflammatory cells and *L*_m_ (*D*) and correlations between PAS positive cells and *L*_m_ (*E*) were analyzed using Spearman correlation. Values are expressed as means ± SE. *P < 0.05, ****P < 0.0001, cigarette smoke (CS) group compared with air group; &*P* < 0.05, CS + LPS group compared with LPS group; Air + vehicle: *n* = 7 animals; air + LPS: *n* = 7 animals; CS + vehicle: *n* = 8 animals; CS + LPS: *n* = 7 animals.

Inflammatory cell infiltration and mucus-producing cells were quantified in H&E- and PAS-stained lung tissue after air or cigarette smoke exposure with or without LPS treatment. Representative histological pictures are depicted in Fig. 2*A*. Exposure to only LPS slightly increased the number of inflammatory cells (Fig. 2*B*) and mucus-producing cells in the lungs (Fig. 2*C*), although this was not significantly different compared with air-exposed mice. Exposure to cigarette smoke significantly increased the number of inflammatory (Fig. 2*A*, red arrows in H&E-stained lung tissue) and mucus-producing cells (Fig. 2*A*, red arrow in PAS-stained lung tissue) (*P* < 0.05 and *P* < 0.0001; Fig. 2, *B* and *C*) compared with air-exposed mice. LPS administration in cigarette smoke-exposed mice did not additionally affect the inflammatory and mucus-producing cell numbers in the lungs (Fig. 2, *B* and *C*). The mean linear intercept (*L*_m_), a measure of interalveolar wall distance representing lung tissue damage, was significantly increased after cigarette smoke with or without LPS administration (32) and was positively correlated with the inflammatory cell infiltration (*r* = 0.5689, *P* = 0.0013; Fig. 2*D*) and the number of mucus-producing cells in the lungs (*r* = 0.5276, *P* = 0.0033; Fig. 2*E*).

#### Inflammatory markers in BAL fluid.

**Figure 3.**
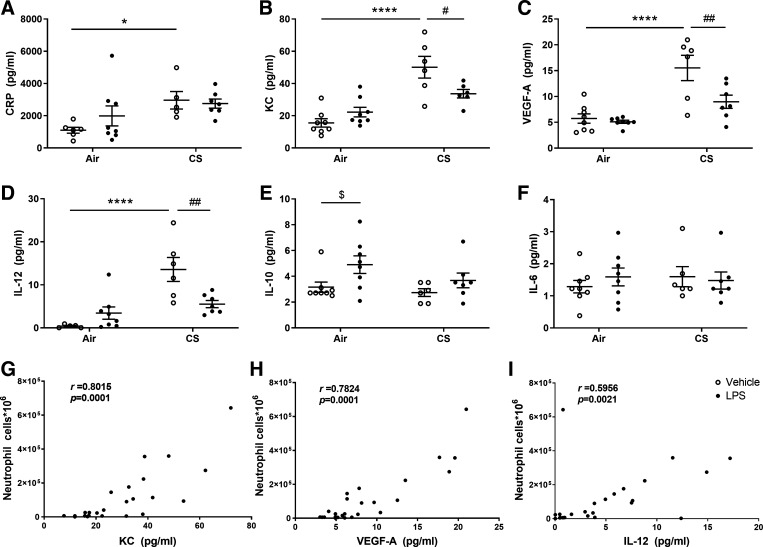
Inflammatory markers in bronchoalveolar lavage (BAL) fluid. Mice were exposed to air or cigarette smoke for 72 days, except on *days 42*, *52*, and *62*. On these days, mice were treated with saline or lipopolysaccharide (LPS) via intratracheal instillation. BAL fluid was collected, and the concentration of inflammatory mediators was determined. The levels of C-reactive protein (CRP; *A*), keratinocyte-derived chemokine (KC; *B*), VEGF-A (*C*), IL-12 (*D*), IL-10 (*E*), and IL-6 (*F*) were determined by using an ELISA and Milliplex Luminex assay kit as described in materials and methods. Values are expressed as means ± SE. *P < 0.05, ****P < 0.0001, cigarette smoke (CS) group compared with air group; $*P* < 0.05, LPS group compared with vehicle group; #*P* < 0.05, ##*P* < 0.01, CS + LPS group compared with CS group; the correlation between KC, VEGF-A, IL-12, and the number of neutrophils in BAL fluid (*G*, *H*, and *I*) were analyzed by using Spearman correlation. Air + vehicle: *n* = 8 animals; air + LPS: *n* = 8 animals; CS + vehicle: *n* = 6 animals; CS + LPS: *n* = 7 animals.

Inflammatory markers were measured in BAL fluid to further confirm lung inflammation after air or cigarette smoke exposure with or without LPS administration. Administration of LPS alone increased IL-10 levels in the BAL fluid compared with air-exposed mice (*P* < 0.05; Fig. 3*E*). However, exposure to cigarette smoke significantly increased the levels of CRP, KC, VEGF-A, and IL-12 in the BAL fluid compared with air-exposed mice (*P* < 0.05 or *P* < 0.001; Fig. 3, *A*–*D*). Cigarette smoke exposure plus LPS treatment decreased the KC, VEGF-A, and IL-12 levels in BAL fluid compared with cigarette smoke treatment alone (*P* < 0.05 or *P* < 0.01; Fig. 3, *B*–*D*). IL-6 levels in BAL fluid were not altered by any treatment (Fig. 3*F*). Significant positive correlations between neutrophil numbers and KC, VEGF-A, and IL-12 levels were demonstrated in the BAL fluid (*r* = 0.8015, *P* = 0.0001; *r* = 0.7824, *P* = 0.0001; *r* = 0.5956, *P* = 0.0021; Fig. 3, *G*–*I*).

### Intestinal Responses

#### Morphology of the small intestines.

**Figure 4.**
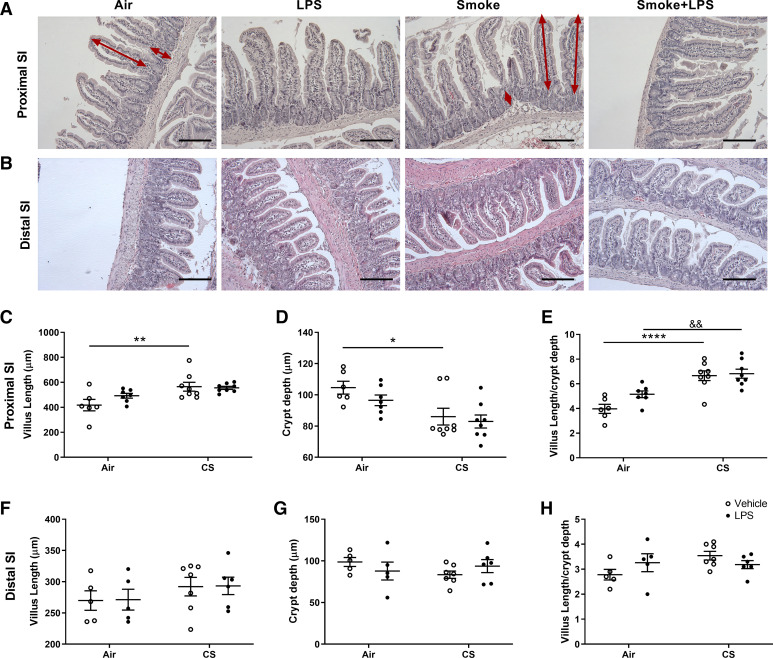
Morphology of the small intestines. Mice were exposed to air or cigarette smoke for 72 days, except on *days 42*, *52*, and *62*. On these days, mice were treated with saline or lipopolysaccharide (LPS) via intratracheal instillation. Intestinal samples were collected, fixed in formalin, and embedded in paraffin. Proximal (*A*) and distal (*B*) small intestinal samples from different treatment groups were stained with hematoxylin and eosin (H&E). Villus length (*C* and *F*) and crypt depth (*D* and *G*) were quantified in the proximal and distal small intestine and villus length/crypt depth ratio was calculated (*E* and *H*) (magnification ×200, Scale bar = 200 μm). Values are expressed as means ± SE. *P < 0.05, **P < 0.01, ****P < 0.0001, cigarette smoke (CS) group compared with air group; &&*P* < 0.01, CS + LPS group compared with LPS group; Air + vehicle: *n* = 6 animals; air + LPS: *n* = 7 animals; CS + vehicle: *n* = 8 animals; CS + LPS: *n* = 8 animals.

The effect of air or cigarette smoke exposure with or without LPS administration on histopathological characteristics of the small intestine was examined as well. Representative histological pictures of the proximal and distal small intestines are depicted in Fig. 4, *A* and *B*, respectively. LPS alone showed no effect on the histomorphology of the small intestines when compared with air-exposed mice (Fig. 4, *C*–*H*). Cigarette smoke exposure increased the villus length (*P* < 0.01; Fig. 4*C*) and decreased the crypt depth (*P* < 0.05; Fig. 4*D*) of the proximal small intestine compared with the air-exposed mice (Fig. 4*A*, red arrows in H&E-stained intestinal tissue). The morphological change is represented in the significantly increased villus length to crypt depth ratio in cigarette smoke-exposed mice (*P* < 0.0001; Fig. 4*E*). LPS administration did not induce any additional effect in cigarette smoke-exposed mice, since no significant changes were observed between cigarette smoke-exposed mice and cigarette smoke-exposed mice with the additional LPS treatment (Fig. 4*E*). No significant changes in the villus length, crypt depth, and villus length to crypt depth ratio in the distal small intestine after cigarette smoke exposure and/or LPS treatment have been demonstrated (Fig. 4, *F*–*H*).

#### SCFAs in cecum content.

The concentrations of SCFAs in the cecum were measured to determine the metabolic activity of the intestinal microbiota. Administration of LPS alone had no significant effect on SCFAs levels in cecum content. The levels of total (iso-) SCFAs, acetic acid, propionic acid, butyric acid, iso-butyric acid, valeric acid, and iso-valeric acid were also not significantly changed after CS exposure (Supplemental Fig. S1, *A*–*H*). Statistically significant effects were only visible for valeric acid and iso-valeric acid when cigarette smoke exposure combined with LPS was compared with the LPS alone group (*P* < 0.01, Supplemental Fig. S1*C*; and *P* < 0.05, Supplemental Fig. S1*D*, respectively). In addition, the total iso-SCFAs were tended to be decreased by CS exposure combined with LPS when compared with the LPS only group (*P* = 0.0529; Supplemental Fig. S1*B*).

#### RNA-sequencing, fecal sIgA, and IgA expression.

**Figure 5.**
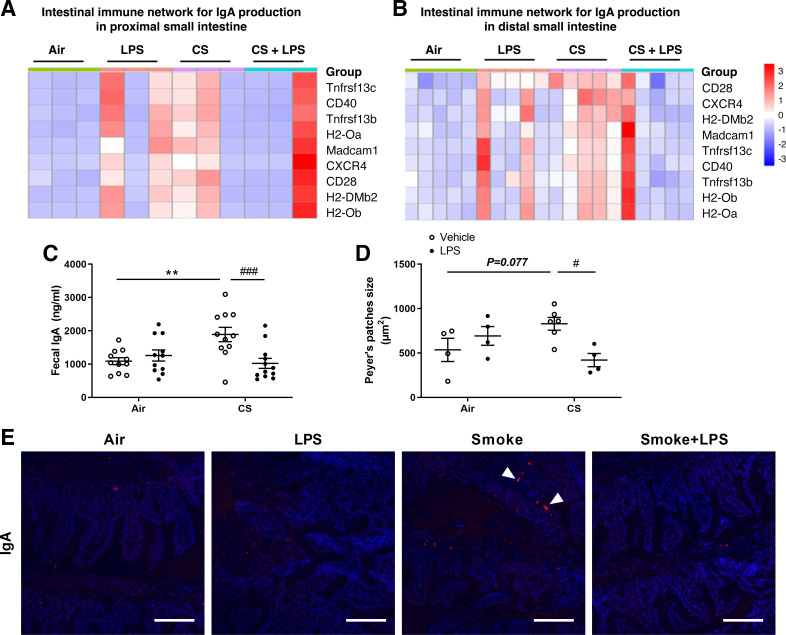
RNA-Sequencing, fecal sIgA, and IgA expression. Mice were exposed to air or cigarette smoke for 72 days, except on *days 42*, *52*, and *62*, on these days mice were treated with saline or lipopolysaccharide (LPS) via intratracheal instillation. Total RNA was isolated and extracted from proximal and distal small intestine tissue. Heat map depicting the scaled gene expression changes in the intestinal immune network for IgA production signaling pathways in the proximal (*A*) and distal (*B*) small intestine. Expression levels of biological replicates were compared (*n* = 3 animals for proximal small intestine or *n* = 5 animals for distal small intestine/group). Color scale of heatmaps represents *Z*-score. Fecal samples were collected and SIgA levels were measured, Air + vehicle: *n* = 11 animals; air + LPS: *n* = 11 animals; CS + vehicle: *n* = 11 animals; CS + LPS: *n* = 12 animals (*C*). Distal small intestinal samples from different treatment groups were collected, fixed in formalin, and embedded in paraffin. The size of the Peyer’s patches was determined in hematoxylin and eosin (H&E)-stained slides from the distal small intestine, Air + vehicle: *n* = 4 animals; air + LPS: *n* = 4 animals; CS + vehicle: *n* = 6 animals; CS + LPS: *n* = 4 animals (*D*). Values are expressed as means ± SE. **P < 0.01, cigarette smoke (CS) group compared with air group; #*P* < 0.05, ###*P* < 0.001, CS + LPS group compared with CS group. Representative images of an immunofluorescent staining with anti-IgA (red) and DAPI (blue) in the distal small intestine are depicted (*E*) (Magnification ×250, Scale bar = 100 μm). CD28, cluster of differentiation 28; CD40, cluster of differentiation 40; CXCR4, C-X-C chemokine receptor type 4; H2-DMb2, histocompatibility 2; H2-Oa, histocompatibility 2, O region alpha locus; H2-Ob: Histocompatibility 2, O region beta locus; Madcam1, mucosal vascular addressin cell adhesion molecule 1; Tnfrsf13b, tumor necrosis factor receptor superfamily 13b; Tnfrsf13c, tumor necrosis factor receptor superfamily 13c.

RNA-Sequencing was performed to detect transcriptome changes in the proximal and distal small intestines in the different treatment groups. KEGG analysis (settings: *P*_adj_ < 0.05 and fold change > 1) indicates that differentially expressed genes were highly enriched in signaling pathways related to the intestinal immune network for IgA production in the distal small intestine of cigarette smoke-exposed mice (Supplemental Fig. S4). A heat map visualization of the gene expression in intestinal samples from different treatment groups is displayed in Fig. 5, *A* and *B*. After cigarette smoke exposure, alterations were observed in the genes associated with the immune network involved in IgA production signaling pathways in the distal small intestine (genes fold change > 1) (Fig. 5*B*), whereas these alterations were less obvious (more variation within groups) in the proximal small intestine (Fig. 5*A*). The changes in genes related to the intestinal immune network for IgA production were supported by increased sIgA levels in fecal samples of cigarette smoke-exposed mice (*P* < 0.01; Fig. 5*C*). Conversely, cigarette smoke exposure plus LPS treatment decreased the fecal sIgA levels (*P* < 0.001; Fig. 5*C*) compared with cigarette smoke only. In addition, an immunofluorescent staining for IgA in the distal small intestine confirmed this IgA expression pattern (Fig. 5*E*, white arrow). The negative control showed no staining in the absence of the primary antibodies (Supplemental Fig. S5). IgA induction primarily occurs in the intestinal Peyer’s patches, the immune sensors of the intestine, and enlargement of Peyer’s patches indicates the induction of adaptive immune responses (37). For this reason, the size of the Peyer’s patches was determined as well. Similarly, cigarette smoke exposure tended to enlarge the size of the Peyer’s patches in the distal small intestine (*P* = 0.077; Fig. 5*D*), whereas cigarette smoke exposure plus LPS treatment decreased the size of the Peyer’s patches (*P* < 0.05; Fig. 5*D*).

### Systemic Inflammation

#### Inflammatory markers in serum.

**Figure 6.**
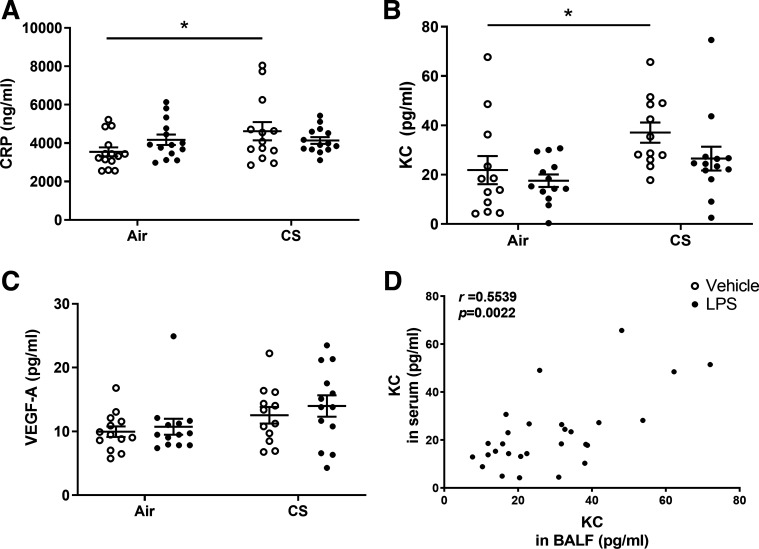
Inflammatory markers in serum. Mice were exposed to air or cigarette smoke for 72 days, except on *days 42*, *52*, and *62*. On these days mice were treated with saline or lipopolysaccharide (LPS) via intratracheal instillation. Serum was collected and the concentration of inflammatory mediators was determined. C-reactive protein (CRP; *A*), keratinocyte-derived chemokine (KC; *B*), and VEGF-A (*C*) levels were detected by using an ELISA and Milliplex Luminex assay kit, respectively. Values are expressed as means ± SE. *P < 0.05; cigarette smoke (CS) group compared with air group. Correlations between KC levels in bronchoalveolar lavage (BAL) fluid and KC levels in serum (*D*) were analyzed by using Spearman correlation. Air + vehicle: *n* = 12 animals; air + LPS: *n* = 13 animals; CS + vehicle: *n* = 12 animals; CS + LPS: *n* = 13 animals.

Inflammatory markers were measured in the serum to investigate systemic inflammation after air or cigarette smoke exposure with or without LPS administration. Administration of LPS alone did not change CRP or KC levels in serum (Fig. 6, *A* and *B*), whereas exposure to cigarette smoke significantly enhanced the levels of both CRP (*P* < 0.05; Fig. 6*A*) and KC in serum (*P* < 0.05; Fig. 6*B*) when compared with air-exposed mice. Although LPS treatment reduced the cigarette smoke-induced KC and CRP levels in the blood, the effect was not statistically significant. The serum level of VEGF-A was not significantly changed in any treatment group (Fig. 6*C*). There was a significant positive correlation between the level of KC in BAL fluid and the amount of KC in serum (*r* = 0.5539, *P* < 0.0022; Fig. 6*D*). Other cytokines in serum, including IL-12, IL-10, and IL-6, were below detection limit (data not shown).

#### T cell subsets in the spleen.

**Figure 7.**
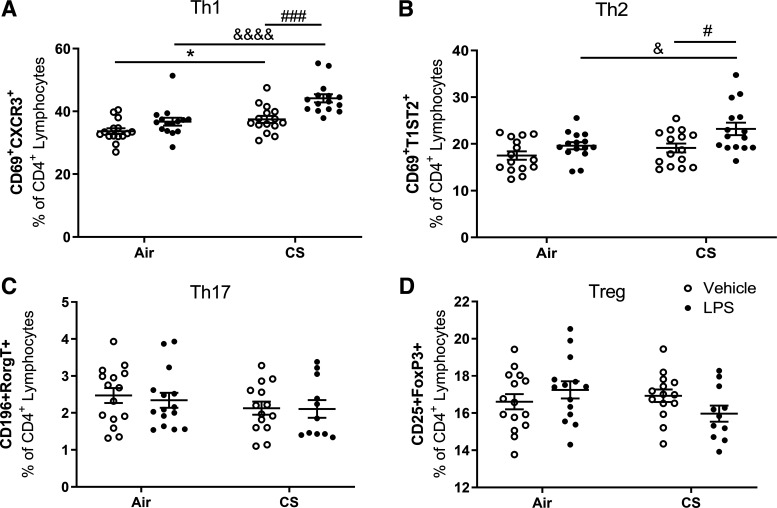
T-cell subsets in the spleen. Mice were exposed to air or cigarette smoke for 72 days, except on *days 42*, *52*, and *62*. On these days mice were treated with saline or lipopolysaccharide (LPS) via intratracheal instillation. Spleens were collected and lymphocytes were isolated and analyzed by flow cytometry for activated Th1 (*A*), Th2 (*B*), Th17 (*C*), and Treg (*D*) cell phenotypes. Values are expressed as means ± SE. *P < 0.05, cigarette smoke (CS) group compared with air control group; &*P* < 0.05, &&&&*P* < 0.0001; CS + LPS group compared with LPS group; #*P* < 0.05, ###*P* < 0.001, CS + LPS group compared with CS group; Air + vehicle: *n* = 15 animals; air + LPS: *n* = 15 animals; CS + vehicle: *n* = 15 animals; CS + LPS: *n* = 15 animals.

Flow cytometric analysis of Th1, Th2, Th17, and Treg cells in the spleen was performed to investigate the effect of air or cigarette smoke exposure with or without LPS administration on T cell subsets in the spleen. Administration of LPS alone had no effect on the different T cell subsets in the spleen when compared with air-exposed mice (Fig. 7, *A*–*D*). Exposure to cigarette smoke enhanced the number of activated Th1 cells compared with the air-exposed control mice (*P* < 0.05; Fig. 7*A*). Cigarette smoke exposure plus LPS treatment significantly increased the percentage of activated Th1 and Th2 cells in the spleen compared with the LPS only and cigarette smoke only treatment groups (*P* < 0.05, *P* < 0.001, or *P* < 0.0001; Fig. 7, *A* and *B*). The percentage of activated Th17 (Fig. 7*C*) and Treg cells (Fig. 7*D*) in the spleen was not affected by any treatment.

## DISCUSSION

Intestinal symptoms are highly prevalent among patients with COPD, and it is becoming more clear that the severity of these intestinal symptoms coincides with the severity of the respiratory symptoms (38). However, the connections and relevance of the connections between the lung and gut are still poorly understood in COPD. The intestines contain many important immune cells and tissues, such as GALT. It has been described that BALT and GALT are both solitary organized mucosa-associated lymphoid follicles and these follicle aggregates have common features and are the origin of B cell trafficking to mucosal effector sites (39). A better understanding of the links between the immune system in the intestine, the systemic immune system, and the lungs might provide insight in COPD but additionally might help the development of new concepts for the treatment of COPD and its comorbidities. To date, experimental research with preclinical COPD exacerbation models (CS + LPS or elastase + LPS) has mainly focused on measuring lung inflammation and lung damage (40), whereas research on intestinal immunity is an unexplored area yet. In this study, cigarette smoke exposure with or without intratracheal LPS treatment was used as a novel model for COPD to investigate the effects on systemic inflammation, and intestinal homeostasis by exploring intestinal histomorphology, the SCFA production in the cecal content, and the intestinal immune network for IgA production in the gut.

To confirm a cigarette smoke-induced lung inflammation and lung damage in our COPD model, airway inflammation, mucus hypersecretion, and mean linear intercept were determined (32). Airway inflammation and mucus hypersecretion are known to be one of the underlying pathophysiological processes in COPD (41). In the current study, histological analysis showed that cigarette smoke exposure induced inflammatory cell infiltration and increased the number of mucus-producing cells in the lung compared with the air-exposed control. These findings were supported by damaged alveoli (*L*_m_) (32). Additional LPS triggers [to mimic bacterial (opportunistic) infections] did not modify the inflammatory cell infiltration, mucus-producing cells in the lung, and *L*_m_ values, which was in agreement with the effect on the number of total BAL fluid cells (32). An extended discussion about the observed airway inflammation and the additional effect of LPS (also on the individual inflammatory cell types in BAL fluid, such as macrophages, neutrophils, and lymphocytes) can be found in the study by Pelgrim et al. (32). In addition, future research should demonstrate whether LPS of any other serotype will cause comparable results.

In general, the degree of damaged lung tissue (*L*_m_) was positively correlated with the inflammatory cell infiltration and the number of mucus-producing cells in the lungs, which indicated progressive and irreversible tissue destruction after cigarette smoke exposure. In agreement with the observations of changed neutrophil numbers in BAL fluid (32), the levels of CRP, KC, VEGF-A, and IL-12 in BAL fluid were significantly increased after cigarette smoke exposure compared with air-exposed mice, whereas additional LPS decreased the level of KC, VEGF-A, and IL-12 in cigarette smoke-exposed mice. Since KC is a neutrophil chemoattractant (42), VEGF-A is expressed by neutrophils and regulates angiogenesis (43), and IL-12 is produced by neutrophils and stimulate growth and function of T cells (44), which explains the correlation of the number of neutrophils and KC, VEGF-A, and IL-12 levels in BAL fluid. This decrease in neutrophil numbers and neutrophil-related cytokines in BAL fluid caused by the additional LPS trigger in cigarette smoke-exposed mice might also be related to the protective role of the immune system after repeated stressors, thereby promoting resilience (and thereby preventing tissue damage) (45).

The next step in this study was to investigate the effect of cigarette smoke with or without LPS on intestinal homeostasis, including small intestinal morphology, cecal SCFA levels, and the small intestinal immune network involved in IgA production have been examined.

In the current study, we focused on the effects of the small intestine, which is an important organ in supporting the body’s immune system. Especially, the Peyer’s patches, located within the small intestine, are an important part of the intestinal local immune system (46). In addition, trophologic insufficiency and absorptive dysfunction of small intestine were often found in patients with COPD (47). In our study, mice treated with cigarette smoke showed an increased villus length and a decreased crypt depth (increased villus length to crypt depth ratio) in the proximal small intestines. LPS treatment did not have any additional effect on the villus length and crypt depth. No differences were demonstrated in the distal small intestines. The increased villus length in the proximal small intestine may indicate mucosal adaptation, as this is regularly seen in patients suffering from intestinal failure (48). In addition, the increased villus length in the proximal small intestine also correlated with decreased body weight as observed in patients with intestinal failure (48). In the current study, a decrease in body weight was noticed in cigarette smoke-exposed mice compared with control mice (32). A decreased body weight is also observed in patients with COPD, especially in those characterized as lean “pink puffers” (the emphysematous patients) (49).

The results related to the observed changes in small intestinal morphology caused by cigarette smoke with and without LPS, raised further interest to investigate the microbial production of SCFAs, to get a first indication of relevant changes in the microbiota composition and activity. Gut microbiota interacts with the immune system and plays a crucial role in health and disease (50). SCFAs, produced by intestinal bacteria after fermentation of fibers, are important metabolites for maintaining intestinal homeostasis and regulating immune cell function (51). Although no obvious changes in cecal SCFAs production were observed induced by cigarette smoke or LPS only treatment, total iso-SCFAs, valeric acid, and iso-valeric acid levels were significantly decreased in mice after cigarette smoke exposure plus LPS treatment compared with the LPS treatment only group. However, this effect might be due to the slight increase of total iso-SCFAs, valeric acid, and iso-valeric acid levels induced by LPS treatment. Although we did not find a change in total SCFAs concentrations, in another COPD model, ambient particulate matter exposure induces gut microbial dysbiosis, and as a consequence, a decrease in total SCFA levels was observed (52). Cecal levels of valeric acid were also significantly decreased in cigarette smoke-exposed rats, which was associated with a decreased population of *Bifidobacterium* and an increase in cecum pH (53). A limitation of this study is the lack of information regarding the gut microbiome. Future studies based on the analyses of the gut microbiota are required to better understand the effect of cigarette smoke with and without LPS on the intestinal microbiota composition and activity.

**Figure 8.**
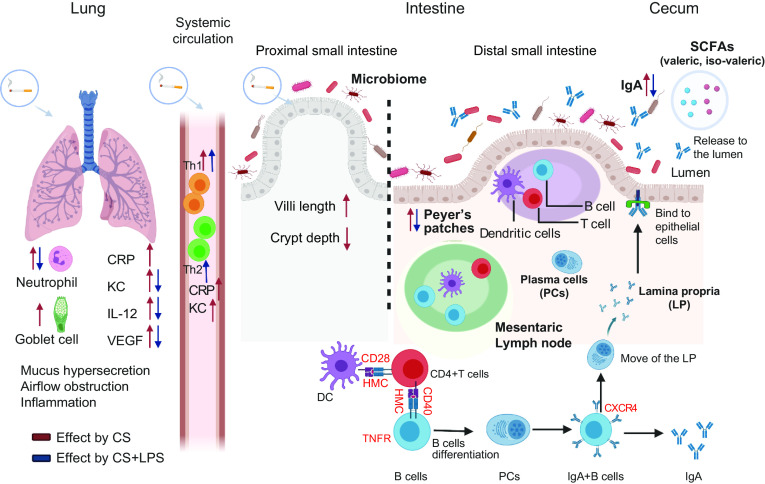
Cigarette smoke exposure leads to lung injury and/or intestinal changes possibly via systemic immune responses. Lung inflammation was induced by cigarette smoke exposure as observed by increased inflammatory cell numbers and PAS-positive cells in lung tissue as well as enhanced mediator release in BAL fluid (CRP, KC, IL-12, and VEGF-A). LPS treatment of cigarette smoke-exposed mice decreased the number of neutrophils and neutrophil-related cytokines (KC, IL-12, and VEGF-A). The interaction between the lung and gut mucosal parts might take place via blood and lymphatic circulation, as observed by increased inflammatory mediators in serum (CRP, KC) and increased Th1 and Th2 cell subsets in the spleen following cigarette smoke exposure. Cigarette smoke exposure plus LPS treatment increased the Th1 and Th2 cell subsets in cigarette smoke-exposed mice. Several intestinal changes were found after cigarette smoke exposure: histomorphological changes in the proximal small intestine, increased cecal SCFAs (possibly microbiota-related changes), stimulation of the intestinal immune network for IgA production, increased fecal IgA levels and increased size of Peyer’s patches in the distal small intestine. Cigarette smoke exposure plus LPS treatment decreased the fecal IgA levels and size of Peyer’s patches. The possibility that soluble compounds from cigarettes enter the blood stream and impact the intestine cannot be excluded, or cigarette smoke may directly enter the gastro-intestinal tract and may (in addition) have local effects on the (intestinal) immune system. The red arrows represent “effects caused by CS” (CS=cigarette smoke). The blue arrows represent “effects caused by CS+LPS.” [Created with BioRender.com.]

IgA (also referred to as sIgA in its secretory form) is known to be a key factor in controlling gut bacterial translocation and development of the mucosal immune system (54, 55). IgA is a hallmark feature of mucosal tissues, of which Peyer’s patches serve as the prototypical model, in which mainly B cells contribute to this mucosal IgA response (56). In the current study, sIgA levels in fecal samples were significantly increased after cigarette smoke exposure, which was confirmed by increased expression of IgA in the distal small intestine of cigarette smoke-exposed mice as observed by an immunofluorescent staining. Interestingly, RNA-Sequencing revealed that the most altered genes in the distal small intestine are involved in the intestinal immune network for IgA production signaling pathways. The changed gene expression may be the result of changes in the differentiation process of the B cells and interactions between B cells, T cells, and dendritic cells after cigarette smoke exposure (or due to changes in the microbiota composition caused by cigarette smoke exposure) (54, 57), possibly leading to higher IgA production. Striking is the finding that changes were observed in the distal small intestine, but not in the proximal small intestine. This might be partly due to the higher density of bacteria in the distal small intestine (18) and the role of gut microbiota in regulating the immune homeostasis in the intestine (58). IgA induction primarily occurs in Peyer’s patches, and the size of Peyer’s patches tended to be enlarged after cigarette smoke exposure in this study. We hypothesize that the changed composition of the microbiota (and related SCFAs) and/or chronic systemic inflammation observed after cigarette smoke exposure can stimulate the IgA production pathway (Fig. 8). Different findings highlight the increased risk of IBD in patients suffering from COPD (7, 9). Interestingly, patients with Crohn’s disease also typically display elevated systemic inflammation and increased systemic antimicrobial IgA responses (59, 60).

An additional effect of LPS was not observed on the intestinal host immunity in the current study. In fact, cigarette smoke exposure plus LPS administration decreased fecal sIgA levels and IgA expression, and reduced the size of the Peyer’s patches in the distal small intestine compared with the cigarette smoke-exposed only group. More research is needed to unravel the mechanism behind the immunosuppressive effect of LPS when combined with cigarette smoke.

One explanation for the gut-lung interaction observed in this study following cigarette smoke exposure can be caused by the initial, localized pulmonary inflammatory response “spill over” and triggering the observed systemic inflammation. The systemic inflammation may affect other immune-related organs, like the spleen and intestine, and hence may disturb intestinal homeostasis and immunity.

The systemic inflammation present in many patients with COPD is correlating with the regulation of inflammation in other tissues and organs, including the intestine, adipose tissue, and spleen (61–63). Blood serum CRP is mostly used as a clinical marker of (acute) systemic inflammation (64). Increased CRP levels in the circulation are associated with poor lung function, systemic comorbidities, worse quality of life, and a higher late mortality in patients with COPD (65, 66). Previous clinical studies showed that systemic inflammation in patients with COPD was associated with increased blood CRP levels and neutrophil cell counts (67, 68). In the current study, cigarette smoke exposure increased serum CRP levels and KC levels and the serum KC levels positively correlated with the KC concentration in BAL fluid. It might be possible that inflammatory mediators leak from the pulmonary to the systemic compartments. Additional LPS treatment did not significantly change the cigarette smoke-induced increase in serum CRP, which was in agreement with the observation in BAL fluid. The decrease in KC serum levels observed by the additional LPS treatment was not significantly different compared with cigarette smoke exposure-only group, but a significant LPS-induced decrease in KC levels in BAL fluid was observed.

Systemic inflammation was also examined by T cells in the spleen. The present results showed that cigarette smoke exposure increased the percentage of Th1 cells in the spleen, while additional LPS treatment further increased the percentage of activated Th1 cells, which might be related to poor prognosis in patients with COPD due to the role of systemic Th1 cells in promoting the inflammatory response (69). Different studies pointed out that increased expression of Th1 cytokines during exacerbations are related to poor prognosis in patients with COPD (69, 70). This was not in agreement with the findings in the current study where the additional LPS treatment did not (yet) affect the *L*_m_ values and decreased the neutrophil influx in the lungs. However, an increased number of macrophages in BAL fluid was observed after the additional trigger with LPS in cigarette smoke-exposed mice as compared with cigarette smoke-exposed mice. Interestingly, cigarette smoke exposure in combination with LPS increased the percentage of activated Th2 cells compared with cigarette smoke-exposed only mice. This result aligns with reports of patients with COPD with acute exacerbations due to bacterial infections that have more Th2 cells in their peripheral blood than those without infection (70, 71). LPS-only treatment also caused an increase in IL-10 in BAL fluid, which is mainly produced by macrophages, Th2, and Treg cells. This is in line with the increased number of macrophages in the BAL fluid after LPS exposure in air-exposed mice (32).

Besides our findings related to systemic inflammation, which may explain the gut-lung interaction, we cannot exclude a direct effect of soluble compounds from cigarette smoke that enter the blood stream and affect the intestine (72). Alternatively, due to the mucociliary clearance of the lungs or direct swallowing of cigarette smoke (73), the cigarette smoke particles may end up in the esophagus and enter the gastrointestinal tract, and possibly have local effects on the immune system along the way (Fig. 8).

In summary, this murine COPD model clearly demonstrated gastrointestinal changes after cigarette smoke exposure, including changes in histomorphology, small fluctuations in cecal SCFA levels, the intestinal immune network related to mucosal IgA production, and size of Peyer’s patches, one of the most relevant mucosal immune organs (Fig. 8). Hence, future COPD research should not only focus on the lung as the target organ but a broader perspective related to other organs, such as the intestines, should be considered. In the future, therapeutical strategies for patients with COPD might therefore not only be directed to the lung, but also to the intestine (gut-lung axis), to decrease systemic disease progression and related comorbidities, such as mental diseases as indicated by Pelgrim et al. (2) (lung-gut-brain axis).

## DATA AVAILABILITY 

Data will be made available upon reasonable request.

## SUPPLEMENTAL DATA

10.6084/m9.figshare.17082590.v1 Supplemental Figs. S1–S5: https://doi.org/10.6084/m9.figshare.17082590.v1.

## GRANTS

This work has been supported by the Top Sector Life Sciences & Health-Top Consortia for Knowledge and Innovation (SLH-TKI)-Lung Foundation Netherlands Public-Private Partnership (PPP) allowance 10.2.16.119. Research grant funding was received from the Chinese Scholarship Council, Award No. 201706170055 (to L. Wang).

## DISCLOSURES

J. Garssen is a part-time employee of Danone Nutricia Research, Utrecht, The Netherlands. A. van Helvoort is an employee of Danone Nutricia Research, Utrecht, The Netherlands. None of the other authors has any conflicts of interest, financial or otherwise, to disclose.

## AUTHOR CONTRIBUTIONS

L.W., C.E.P., A.v.H., A.D.K., G.F., and S.B. conceived and designed research; L.W., C.E.P., L.N.P.M., S.K., I.v.A., and T.L.-M. performed experiments; L.W. and C.E.P. analyzed data; L.W., P.A.J.H., G.F., and S.B. and interpreted results of experiments; L.W. prepared figures; L.W. drafted manuscript; L.W., C.E.P., L.N.P.M., S.K., I.v.A., T.L.-M., A.v.H., A.K., A.D.K., J.G., P.A.J.H., G.F., and S.B. edited and revised manuscript; L.W., C.E.P., L.N.P.M., S.K., I.v.A., T.L.-M., A.v.H., A.K., A.D.K., J.G., P.A.J.H., G.F., and S.B. approved final version of manuscript.
